# An Enhanced Insect Pest Counter Based on Saliency Map and Improved Non-Maximum Suppression

**DOI:** 10.3390/insects12080705

**Published:** 2021-08-06

**Authors:** Qingwen Guo, Chuntao Wang, Deqin Xiao, Qiong Huang

**Affiliations:** 1 College of Mathematics and Informatics, South China Agricultural University, Guangzhou 510642, China; guoqingwen@stu.scau.edu.cn (Q.G.); deqinx@scau.edu.cn (D.X.); qhuang@scau.edu.cn (Q.H.); 2 Guangzhou Key Laboratory of Intelligent Agriculture, Guangzhou 510642, China

**Keywords:** insect pest counting, deep learning, saliency map, non-maximum suppression, tune-up box

## Abstract

**Simple Summary:**

Simple Summary: Accurately counting the number of insect pests from digital images captured on yellow sticky traps remains a challenge in the field of insect pest monitoring. This paper develops a new approach to counting the number of insect pests using a saliency map and improved non-maximum suppression. Specifically, a saliency map is exploited to construct a region proposal generator, and a convolutional neural network (CNN) model is used to classify each region proposal as a specific insect pest class, resulting in detection bounding boxes. An improved non-maximum suppression is further developed to sophisticatedly handle the redundant detection bounding boxes, and the insect pest number is thus obtained through counting the handled detection bounding boxes, each of which covers one insect pest. As this insect pest counter may miscount insect pests that are close to each other, the widely used Faster R-CNN is further integrated with the mentioned insect pest counter to construct a dual-path network. Extensive experimental simulations show that the two proposed insect pest counters achieve significant improvements in terms of F1 score against state-of-the-art object detectors as well as insect pest detection methods.

**Abstract:**

Accurately counting the number of insect pests from digital images captured on yellow sticky traps remains a challenge in the field of insect pest monitoring. In this study, we develop a new approach to counting the number of insect pests using a saliency map and improved non-maximum suppression. Specifically, as the background of a yellow sticky trap is simple and the insect pest object is small, we exploit a saliency map to construct a region proposal generator including saliency map building, activation region formation, background–foreground classifier, and tune-up boxes involved in region proposal generation. For each region proposal, a convolutional neural network (CNN) model is used to classify it as a specific insect pest class, resulting in detection bounding boxes. By considering the relationship between detection bounding boxes, we thus develop an improved non-maximum suppression to sophisticatedly handle the redundant detection bounding boxes and obtain the insect pest number through counting the handled detection bounding boxes, each of which covers one insect pest. As this insect pest counter may miscount insect pests that are close to each other, we further integrate the widely used Faster R-CNN with the mentioned insect pest counter to construct a dual-path network. Extensive experimental simulations show that the two proposed insect pest counters achieve significant improvement in terms of $F_{1}$ score against the state-of-the-art object detectors as well as insect pest detection methods.

## 1. Introduction

Most crop growers face challenges from numerous agricultural insect pests, which may result in a reduction in crop production. To tackle this problem, integrated insect pest management has been developed and applied in practice. A strong integrated insect pest management system can help farmers apply appropriate treatments to crops and consequently reduce their economic losses. For example, as a crucial component in the integrated insect pest management, monitoring the number of insect pests can assist crop growers to spray the correct amount of pesticides on the correct field.

According to the literature, three methods can be used to obtain the number of insect pests in the field: (i) manual observation; (ii) using multi-dimensional data (e.g., soil temperature and leaf wetness) to estimate the order of magnitude of the insect pest number [1,2]; and (iii) capturing insect pest images with trapping devices followed by counting the insect pest numbers via computer-vision-based detection. The first method is too expensive and slow, and the second cannot output an exact value. In contrast, the third one overcomes the disadvantages of the first two methods and thus has become the main research direction of insect pest monitoring.

In the past decades, computer-vision-based insect pest detection has significantly advanced [3,4,5]. Some researchers applied support vector machine using a sliding window to detect planthoppers in paddy fields [3,4]. Combined with the bag-of-features model, support vector machine was previously used to recognize vegetable insect pests [5]. Although support vector machine is a popular machine learning technique, it requires careful engineering and considerable domain expertise for the design of the feature extractor. This hinders its application in practical crop production scenarios.

To overcome the limitations of conventional support vector machine, the emerging deep learning techniques can learn features automatically in an end-to-end manner. As a result, they have been widely applied in smart agriculture as well as other fields [6], producing remarkable breakthroughs [6,7,8,9,10,11,12,13,14,15,16,17,18,19,20,21,22,23,24,25,26,27,28,29,30,31,32,33,34]. A brief introduction to these approaches is presented in the next section.

For the automatic monitoring of insect pests, researchers generally install sticky traps in the field to monitor insect pests, deploy a camera to obtain images of the installed sticky traps, and finally apply deep-learning-based object detectors to conduct insect pest detection and number counting, as illustrated in [35]. Although desirable performance has been achieved, two problems are experienced by most state-of-the-art deep-learning-based insect pest detectors. In particular, most deep-learning-based insect pest detectors pay excessive attention to irrelevant background regions during the training phase and thus cannot accurately identify the beneficial and neutral insects from the insect pests in the testing phase. In addition, the non-maximum suppression (NMS) algorithm used in the existing deep-learning-based insect pest detectors ignores the relationship between detection bounding boxes; consequently, a false positive with a higher confidence is generally used to suppress a number of true positives that correspond to the same target but have smaller confidences.

To address these two problems, we developed a new insect pest counter using a saliency map and improved NMS (INMS). Specifically, as most easy negatives, such as background regions, are simple and humans are generally attracted by saliency components in images, we then exploited a saliency map [24,36,37] to filter out most easy negatives of background regions, which in turn would guide the deep-learning-based detector to pay more attention to the hard negatives, such as the beneficial and neutral insects, and thus improve the detection accuracy. In addition, by considering the relationship between detection bounding boxes, we used a merging strategy arising from OverFeat [13] to integrate the candidate detection bounding boxes with small confidence with those with high confidence, which consequently enhance the robustness of the conventional NMS. By combining the saliency-map-based region proposal generator and the INMS with the conventional general-purpose object detector, we thus constructed a new scheme for insect pest detection and counting. Moreover, as the developed insect pest counter may miscount insect pests that are close to each other, we further integrated the developed insect pest counter with the widely used Faster R-CNN [15], yielding another insect pest counter with dual-path networks.

To illustrate the feasibility of the proposed scheme, we applied it to count the diamondback moth, which is a key insect pest of vegetables. Extensive experimental simulations showed that our two proposed insect pest counters achieve remarkable improvement in terms of $F_{1}$ score over the state-of-the-art deep-learning-based object detectors. The dual path network based scheme provides remarkable improvement over the other considered method, and the constructed insect pest counter provides a significant improvement compared to the state-of-the-art insect pest detection method. These findings demonstrate the feasibility and effectiveness of the proposed scheme in monitoring insect pest numbers.

The main contributions of this study are three-fold:

- According to characteristics of yellow sticky traps, the saliency map, background–foreground classifier, and tune-up box techniques are used to remove most inessential background regions, which well facilitates the accurate detection of insect pests.
- A merging strategy is developed to improve the conventional NMS that is adopted in most deep-learning-based insect pest detectors.
- Two saliency-map- and INMS-based insect pest counters are constructed. Extensive simulations of the diamondback moth show that the developed counters achieve a desirable detection rate (DR) with a significantly lower false DR (FDR), providing a significant improvement in terms of $F_{1}$ score over most state-of-the-art deep-learning-based object detectors and the state-of-the-art insect pest detection methods.

For convenience, Table 1 summarizes the main abbreviations in this paper. The rest of this paper is organized as follows: Section 2 introduces related works in the literature. Section 3 presents the experimental dataset and proposes an alternative insect pest counter. An enhanced dual-path-network-based version is presented in Section 4. Experimental results and analyses are outlined in Section 5. Section 6 draws the conclusion to this study.

## 2. Related Works

### 2.1. CNN-Based Object Detectors

Among deep-learning techniques, the convolutional neural network (CNN) is the one predominantly used for computer vision tasks. All CNN-like models have their roots in neocognitron, and their architectures are somewhat similar to that of the animal visual cortex [7]. These models sophisticatedly integrate four architectural components: local receptive fields, shared weights, pooling layers and hierarchical structures [6]. Details of these components have been provided in previous studies [6,7,8].

Unfortunately, the neocognitron does not have an end-to-end supervised-learning algorithm and is thus imperfect. To deal with this problem, many studies have been conducted. In the 1990s, a CNN model called LeNet was proposed [8]. It uses a backpropagation algorithm as the general-purpose learning procedure and requires little handcrafted engineering. In LeNet, only some hyperparameters need to be fine-tuned (e.g., the network depth and the filter size). Due to LeNet and the backpropagation algorithm, the academic community has established the modern CNN framework and has further proposed several improved variants such as AlexNet [9], GoogLeNet [10], VGG [11] and ResNet [12]. These CNN-like models have achieved record breaking results in object recognition. Recently, the use of graphics processing units has increased the efficiency of CNN. Moreover, some open-source platforms (e.g., TensorFlow [39] and PyTorch [40]) have helped the CNN to gain popularity.

Inspired by the revolution due to CNNs in object recognition, some researchers used the CNN to develop generic object detection methods, which include OverFeat [13], R-CNN [14], Faster R-CNN [15], R-FCN [16], YOLO [17] and SSD [18]. OverFeat uses a sliding-window CNN to detect objects, which has feasible computational complexity since convolution kernels naturally share computations for overlapping regions [13]. R-CNN employs a selective search to generate region proposals and then adopts a CNN to classify them [14]. To tackle the time consumption issue of the selective search in R-CNN, Faster R-CNN uses a region proposal network and *k* anchor boxes to quickly generate region proposals and uses another network to classify the generated region proposals [15]. In essence, Faster R-CNN can be viewed as a two-stage version of OverFeat, in which the first stage is used to discard a large number of easy negatives so that the second stage can focus on handling more difficult negatives [15,19]. Later, R-FCN replaced the costly fully connected layer of Faster R-CNN with a shared region-of-interest subnetwork and position-sensitive score maps to achieve more efficient object detection [16]. YOLO [17] and SSD [18] divide feature maps into grids and use CNNs to detect objects according to contents in grid cells. In contrast to YOLO, which only uses the deepest feature map, SSD uses several feature maps and anchor boxes with respect to the pyramid structure to detect objects of different sizes [17,18].

### 2.2. Agricultural Applications of CNN

Due to the powerful capability of the CNN, it has been increasingly applied in agriculture. For example, Nazri et al. [20] used a thresholding algorithm to separate image pixels into the foreground (i.e., the pest) and background (i.e., the sticky card) and next deployed a CNN to identify the separate foreground. Wang et al. [21] employed transfer-learning-based CNN to identify plant pests. It achieves comparable performance to human beings, illustrating well the adaptability of the CNN to agricultural problems. Later, Rahman et al. [22] constructed an elaborate lightweight CNN to recognize rice diseases and pests, achieving acceptable accuracy with a significantly reduced model size. Recently, Nanni et al. [23] incorporated a saliency map in a CNN model to improve the pest identification performance, where the saliency map was considered a kind of attention mechanism.

CNNs have also been applied to pest counting. It first detects pests in images using bounding boxes and then counts the number of pests. As the counting operation is rather simple, pest detection becomes the key step in pest counting. In the literature, dozens of pest detection approaches have been developed. For instance, Liu et al. [24] used a saliency map and CNN to localize and classify pests, respectively, in a paddy field. Ding and Taylor [25] applied a CNN to build a sliding-window moth detector, obtaining a desirable performance. The principle in [25] is similar to that of OverFeat. In [26], YOLO was employed to detect Asian citrus psyllids. In [27,28,29,30,31,32,33,34,41], Faster R-CNN and its slightly modified versions were used to detect pests. These methods provide accurate performance and thus demonstrate the effectiveness of Faster R-CNN for pest detection.

## 3. Materials and Methods

### 3.1. Dataset

**Figure 1:**
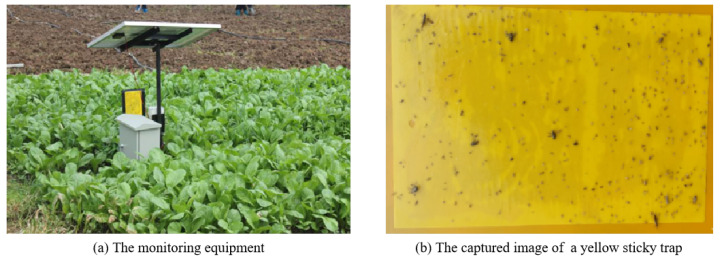
Illustration of (**a**) the monitoring equipment and (**b**) the captured image of a yellow sticky trap.

In our work, we used yellow sticky traps installed in a vegetable field to monitor insect pests, and adopted a digital camera fixed in the designed monitoring equipment to obtain images of yellow sticky traps. Figure 1 illustrates the monitoring equipment and the captured images.

The acquired images were stored in JPEG format with a resolution of 3120 × 4160 pixels. Given the trade-off between performance and computational complexity in model training, we randomly cropped the acquired images to 415×430, 470×250, 415×320, and so forth. In total, we constructed a dataset with 1789 images of yellow sticky traps.

Each image was annotated using the free software, LabelImg [42], by two trained volunteers in entomology. The annotations including insect pest categories and bounding box coordinates were saved as XML files in PASCAL VOC format, where insect pest categories were the diamondback moth and others. In the initial stage, we mainly focused on the diamondback moth, which is a key insect pest in the vegetable field.

In the simulation, we used 650 images as the training set, 50 images as the validation set, and the other 1089 images as the test set.

### 3.2. Methodologies

It is reported that multi-stage object detectors, such as Faster R-CNN, usually perform better than the other kinds of object detectors [19]. Therefore, in this study, we mainly exploited the architecture of multi-stage object detectors to build a more appropriate pest detector for pest counting.

As Faster R-CNN and OverFeat adopt a sliding window to generate candidate regions, a huge imbalance between easy negatives (e.g., regions only containing a patch of the yellow sticky trap) and hard negatives (e.g., regions including a neutral object) would occur. This imbalance would result in the training of the CNN-based object classifier being inefficient because easy negatives tend to overwhelm the training. Although Faster R-CNN employs a region proposal network to eliminate many easy negatives, the region proposal network is implemented in a sliding window fashion, which burdens the classification task with a large number of irrelevant regions.

As the background regions in our scenario were simple and humans are generally attracted by the salient components of a given image, we exploited a saliency map to remove most inessential background regions (i.e., easy negatives), forming the first stage of our proposed scheme. In the second stage, we deployed the widely-used CNN to conduct object classification for each region proposal generated in the first stage, and develop the INMS (i.e., improved non-maximum suppression) to enhance the detection and classification performance.

**Figure 2:**
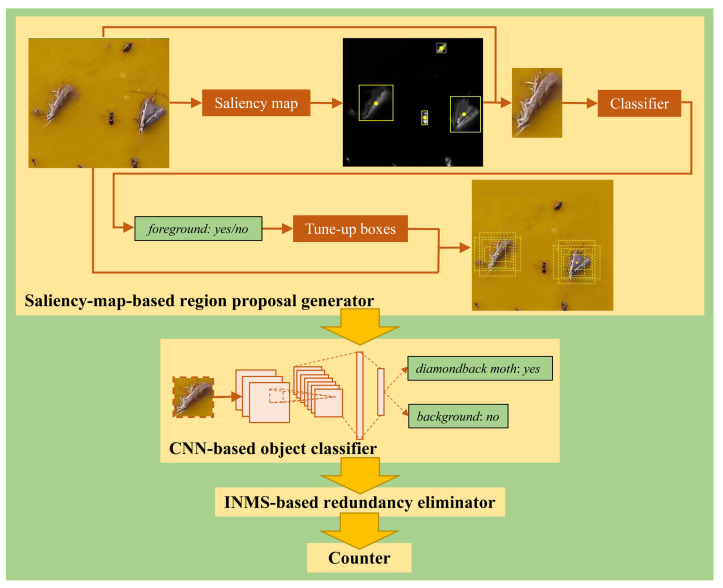
The pipeline of our proposed insect pest counter. It is composed of four modules, i.e., a region proposal generator based on the saliency map, an insect pest classifier based on the CNN, a redundancy eliminator based on INMS, and an insect pest counter.

By following this idea, we constructed a region proposal generator to yield region proposals that probably contain insect pest objects, employed CNN as the classification module, designed an INMS-based module to remove redundant detection bounding boxes, and finally adopted a counter to calculate the number of insect pests on a given yellow sticky trap. The steps form the proposed scheme for insect pest counting, as illustrated in Figure 2. Details of these modules are presented below.

#### 3.2.1. Saliency-Map-Based Region Proposal Generator

As mentioned above, we exploited the saliency map to filter most of the inessential easy negatives. A saliency map consists of saliency components, such as the edges and textures of a given image, which may well mimic the most relevant regions captured by humans’ attention mechanism.

The yellow sticky trap background in our work is rather simple. Thus, we adopted the efficient method in [37], which can be easily implemented, to generate the saliency map as illustrated in Figure 2.

After obtaining the saliency map, we constructed the mask for the saliency map, exploited the mask to produce activation regions, and used a predefined threshold to remove non-target activation regions. Subsequently, we applied the CNN to classify each activation region as the foreground or background followed by imposing tune-up boxes on the foreground region to generate region proposals. These steps form the developed region proposal generator, which are described in detail as follows.

- By following [37], first convert image I of size H×W into LAB color space, yielding one luminance and two color channels: L, a, and b, respectively. Then, generate the saliency map S via the method in [37] as:
  (1) $$S\left(r,c\right)=∥I_{\mu }-\left[I_{\omega_{hc}}^{L}\left(r,c\right),I_{\omega_{hc}}^{a}\left(r,c\right),I_{\omega_{hc}}^{b}\left(r,c\right)\right]{∥}_{2},r\in \left[1,H\right],c\in \left[1,W\right],$$
  where $I_{\mu }$ is a 1×3 vector containing mean values of L, a, and b; $I_{\omega_{hc}}^{o}\left(o\in \left\{L,a,b\right\}\right)$ denotes the Gaussian blurred version of L, a and b, respectively; and ${∥\cdot ∥}_{2}$ is the $L_{2}$ norm.
- Produce a mask with M. That is, if S(r,c) is larger than or equal to a predefined threshold α, M(r,c) is set to be 1; otherwise, M(r,c) is determined as 0.
- Use mask M to generate activation regions a. Specifically, link pixels M(r,c)=1 together to form a connected graph, and then take each connected enclosed graph as an activation region, namely $a=\{\left(x_{\min},y_{\min},x_{\max},y_{\max}\right)\}$, where a∈a.
- Discard the activation region a∈a if its area is below the preset threshold β. This is because these activation regions are usually a small non-target specie (i.e., an other than a diamondback moth).
- Classify each a∈a as the background or foreground using classifier $C_{1}$. Suppose that $b_{\mathrm{gt}}$ denotes the bounding box of the ground-truth object, and then define the iogt as:
  $$\mathrm{iogt}\left(a,b_{\mathrm{gt}}\right)=\frac{\mathrm{area}(a\cap b_{\mathrm{gt}})}{\mathrm{area}(b_{\mathrm{gt}})},$$
  where area(·) is the function computing the given area. If all iogt(a,·) are smaller than the preset threshold, the activation region of *a* is considered a background region; otherwise, it is regarded as a foreground region. The construction of $C_{1}$ is presented in Section 3.2.2.
- Yield region proposals b. Once the activation region *a* is classified as the background, remove *a*; otherwise, add *a* into b, then calculate its central point $\left(x_{c},y_{c}\right)$, and impose *k* tune-up boxes of preset scales with respect to $\left(x_{c},y_{c}\right)$ to b. In essence, $\left(x_{c},y_{c}\right)$ can be considered as an anchor, which would be much less than those generated by Faster R-CNN.

These steps are summarized in Algorithm 1.
**Algorithm 1:** Region proposal generator using a saliency map.
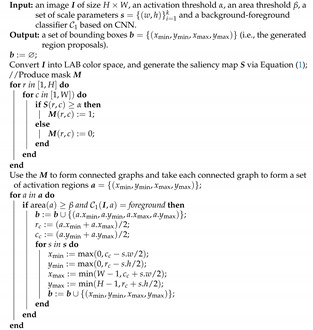


Compared with the region proposal network of Faster R-CNN that takes all sliding windows of the entire image as candidate region proposals, our region proposal generator uses a saliency map to adaptively produce candidate region proposals. This treatment effectively decreases the inessential background regions and thus reduces the computational load in the object classification.

**Figure 3:**
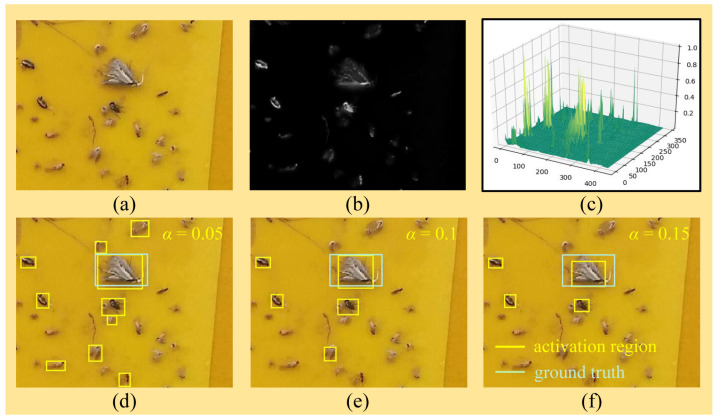
An example of a saliency map. (**a**) The original image; (**b**) the saliency map; (**c**) the three-dimensional diagram, where the z-axis denotes the saliency score; and (**d**–**f**) the saliency map with α = 0.05, 0.1, and 0.15, respectively.

Compared with the method in [24], the developed region proposal generator additionally integrates the background–foreground classifier and tune-up boxes for region proposal generation. The background–foreground classifier facilitates the further removal of small and easy negatives and thus contributes to improving the detection accuracy of the subsequent object classifier. The tune-up boxes help to make a fusion decision and thus prevent the whole detector from resulting in mistaken decisions in only one viewpoint. Moreover, the tune-up boxes also provide the benefits of refining the view field of the object classifier in the second stage and addressing the challenges discussed in [24]: it is hard to properly determine the activation threshold α, that is, a small α may capture too much background noise while a large α cannot well highlight the whole target object, as illustrated in Figure 3.

#### 3.2.2. CNN-Based Background-Foreground and Object Classifiers

As mentioned in Section 3.2.1, the background–foreground classifier $C_{1}$ is used in the region proposal generator to classify an activation region as the background or the foreground. After region proposals are generated, each region proposal is then input to the object classifier $C_{2}$ to determine the category, that is, a diamondback moth or background. Since CNN has powerful classification ability, it is exploited in our method to implement these two classifiers. As both classifiers classify a concerned region into different classes, they can be essentially considered equivalent. Thus, we adopted the same CNN model for both $C_{1}$ and $C_{2}$.

As we mainly aimed to find a feasible CNN model instead of constructing a powerful one, we explored a number of popular CNNs and then choose one with desirable performance as the preferable CNN for $C_{1}$ and $C_{2}$. As most inessential easy negatives were removed by the saliency map, we first considered a lightweight CNN with an acceptable performance and low computational complexity. Table 2 lists a lightweight CNN model, CNN ${}_{lw}$, which was mainly modified from the popular CNNs such as OverFeat, R-CNN and YOLO. CNN ${}_{lw}$ deploys a stride of two and max-pooling because insect pests are small objects and a small stride facilitates the capture of small insect pests.

The CNN model in Table 2 consists of five layers. The first three layers are convolutional layers and the last two are the fully convolutional layers. Each of the first three layers includes convolution and max-pooling parts. The kernel of the max-pooling part is 2×2, and the structure of the convolution part is represented as (k,s,n), where *k*, *s* and *n* denote the size of the convolution kernel, the filter stride, and the number of convolution kernels, respectively. The activation function of the CNN model is the sigmoid function.

Although the aforementioned lightweight CNN provides an acceptable performance with low computational complexity, we also considered deeper CNNs such as VGG [11] to obtain better performance at the cost of acceptable computation time. VGG has two versions, that is, VGG-16 and VGG-19. As the insect pest object in our scenario is small, we adopted the VGG-16 in our work to obtain a suitable receptive field and reduce the computational load as much as possible. The details of VGG-16 are provided in [11].

Although the same CNN structure is adopted for both the $C_{1}$ and $C_{2}$, their hyper-parameters were trained with different data. Classifier $C_{1}$ is trained using the input and output data described in Algorithm 1, whereas the input and output data of classifier $C_{2}$ are formed as follows. As the $C_{2}$ actually classifies a region proposal into a specific category, the input is any region proposal b∈b generated by the region proposal generator (Algorithm 1) and the output is the category label, namely *ℓ*, which is determined below. Specifically, suppose that $b_{\mathrm{gt}}\in b_{\mathrm{gt}}$ denotes a ground-truth bounding box. Then define the intersection-over-union value of *b* and $b_{\mathrm{gt}}$ as

$$\mathrm{iou}\left(b,b_{\mathrm{gt}}\right)=\frac{\mathrm{area}(b\cap b_{\mathrm{gt}})}{\mathrm{area}(b\cup b_{\mathrm{gt}})}.$$

If there is a $b_{\mathrm{gt}}$ such that $\mathrm{iou}(b,b_{\mathrm{gt}})$ is larger than or equal to the predefined threshold, the *ℓ* of *b* is then determined as the category label of $b_{\mathrm{gt}}$.

#### 3.2.3. Improved Non-Maximum Suppression

Via the object classifier $C_{2}$, each region proposal generated by the region proposal generator is classified as one of the target objects or the background. The classified region proposal is then denoted as the detection bounding box. According to Algorithm 1, a number of region proposals may correspond to one insect pest object, and thus some detection bounding boxes may significantly overlap with each other, which causes redundancy. To eliminate redundant detection bounding boxes, the NMS (non-maximum suppression) technique is generally used. Specifically, it first calculates the intersection-over-union between two target detection bounding boxes, say $b_{0}$ and $b_{1}$, then determines them to be overlapped if $\mathrm{iou}(b_{0},b_{1})$ is larger than a predefined threshold, so the one with with higher confidence remains but the other is removed. This process continues until no overlapped detection bounding boxes can be found.

We adopted the conventional NMS algorithm in our pipeline and found that its performance over the validation set was unacceptable because the insect pests in our scenario have large inter-class similarity; thus, mistaken classification occurred with high probability, which is illustrated as follows: Suppose that φ denotes a target insect pest and $b_{0}$ and $b_{1}$ are two detection bounding boxes related to the φ. So, the $b_{1}$ contains the whole φ while the $b_{0}$ contains only part of φ. Due to the high inter-class similarity, $b_{0}$ has higher confidence than $b_{1}$, and $b_{0}$ and $b_{1}$ are categorized as two different kinds of insect pests (i.e., $b_{1}$ is correct, but $b_{0}$ is wrong). In this situation, the conventional NMS suppresses $b_{1}$, which clearly results in error suppression.

By observing that the object classifier could successfully identify the same insect pest at most angles (with respect to *k* tune-up boxes), we improved the conventional NMS by considering the relationship between detection bounding boxes, yielding the INMS (improved NMS). Algorithm 2 presents the INMS, where the numel(·) function calculates the element number of a set. It first sorts the set of detection bounding boxes b in ascending order of the box’s area, and then denote the detection bounding box with the least area as $b_{0}$. Next, compute the intersection-over-union between $b_{0}$ and $b_{i}\in b\left(i=1,2,\ldots \right)$, subsequently seek the first $b_{i}$ that has the same category label as $b_{0}$ and an $\mathrm{iou}(b_{0},b_{i})$ is larger than the predefined threshold α, and finally merge $b_{0}$ in $b_{i}$ followed by resorting the merged result in ascending order of box’ area. If no $b_{i}$ can be found, then $b_{0}$ is thought to have no overlap with the other detection bounding boxes, and thus $b_{0}$ is removed from b and then placed into set t. This process iterates until b is empty. Afterwards, the conventional NMS is imposed on the final set of t and the detection bounding box whose confidence is larger than or equal to the predefined threshold γ is chosen as the INMS-handled result.
**Algorithm 2:** Improved non-maximum suppression using a merging strategy.
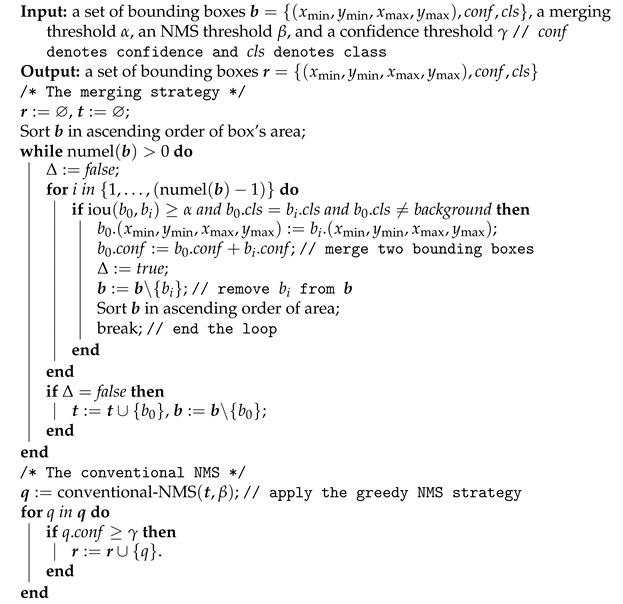


Our INMS is an integration of the conventional NMS and the merging strategy of OverFeat [13]. It overcomes the disadvantages of both NMS and OverFeat. That is, INMS tackles the issue of error suppression in the NMS by considering the relationship between detection bounding boxes. INMS also addresses the difficulty in the determination of the proper threshold γ for the merging process of OverFeat. In more detail, OverFeat merges rather than suppresses the overlapped detection bounding boxes, and then removes the merged detection bounding box, whose confidence is smaller than a present threshold γ. As the confidence of the merged detection bounding box varies widely, a confidence larger than γ may still be a false positive. By involving the suppression of conventional NMS to tackle this problem, the false positives are eliminated by a true value with higher confidence.

#### 3.2.4. Insect Pest Counter

After processing with INMS, the redundant detection bounding boxes are removed. Therefore, each insect pest is labeled by only one detection bounding box. By counting the number of these detection bounding boxes resulting from the INMS module, the number of insect pests can be easily obtained.

## 4. Insect Pest Counter Enhancement Based on Dual Path Network

As described in Section 3, the proposed insect pest counter exploits a saliency map to remove most inessential easy negatives so that the object classifier can better handle the hard negatives. An improved version of NMS was designed to achieve a more accurate detection performance. These will be demonstrated in the experimental section.

**Figure 4:**
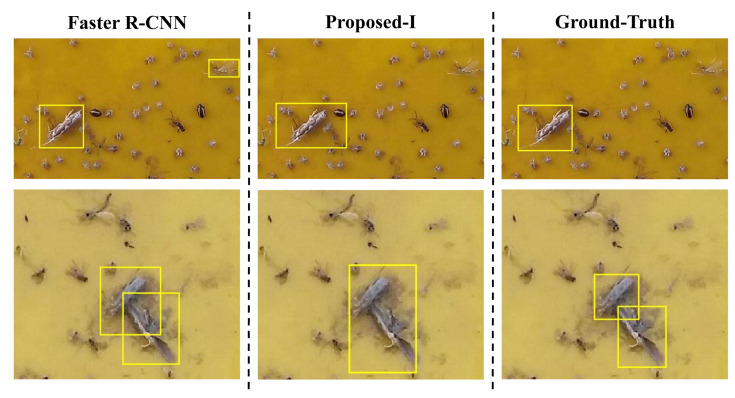
Illustration of the detection and classification results for the proposed scheme and Faster R-CNN over the validation set. The top row is the results of the proposed scheme, and the bottom row is those of Faster R-CNN.

The experimental simulation over the test set, however, showed that the proposed insect pest counter considers insect pests close to each other as one object, which decreases the DR (detection rate) accordingly, as illustrated in Figure 4. We found that, although the proposed scheme can accurately detect insect pests, it mistook two diamondback moths located close to each other as one because the proposed scheme takes a connected graph constructed from the saliency map as an activation region (Algorithm 1). As a result, insect pests sufficiently close to each other are probably connected to form a connected graph; thus, they are determined as one activation region, so some of the insect pests are not counted.

Faster R-CNN deploys a sliding window to generate candidate region proposals followed by inputting all these region proposals to the object classifier to determine the object category. This scheme increases the DR at the cost of increased FDR (false detection rate). As shown in the bottom row of Figure 4, Faster R-CNN successfully detects the diamondback moth that was missed by the proposed scheme. However, as Faster R-CNN generates many more region proposals, it would probably mistake a non-target as a diamondback moth and thus result in false detections.

**Figure 5:**
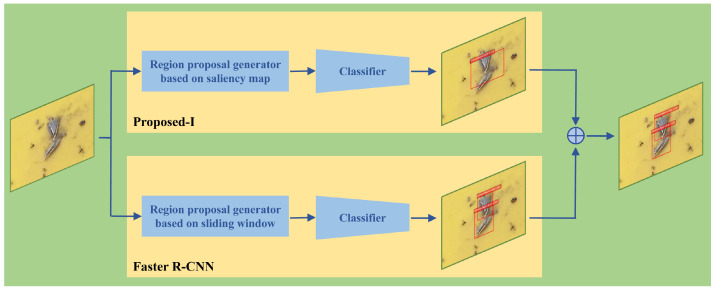
The architecture of the enhanced insect pest counter using the dual-path network. The top path is the proposed network described in Section 3 and the bottom path is Faster R-CNN. ‘⊕’ denotes the fusion module.

In summary, the proposed insect pest counter achieves a lower FDR with a feasible DR, whereas Faster R-CNN obtains a higher DR at the cost of increased FDR. In other words, the proposed counter and Faster R-CNN are complementary to each other. As such, we integrated the proposed scheme in Section 3 and Faster R-CNN to construct an enhanced insect pest counter using the dual-path network, as demonstrated in Figure 5. For notational convenience, the proposed insect pest counter in Section 3 is denoted *Proposed-I* and the enhanced version in this method is called *Proposed-II*.

In Figure 5, the top path depicts the network in Section 3.2 and the bottom path depicts Faster R-CNN, both of which yield the final detection bounding boxes. As these two paths may result in different detection bounding boxes, we finally designed a fusion module to obtain the final detection and classification results. In more detail, if any detection bounding box $bf_{i}\left(i=0,1,\ldots \right)$ generated by Faster R-CNN overlaps that $bp_{j}\left(j=0,1,\ldots \right)$ yielded by the proposed network in Section 3.2, that is, $\mathrm{iou}(bf_{i},bp_{j})>0$, then $bf_{i}$ is a reasonable detection bounding box and is retained; otherwise, $bf_{i}$ is considered to be a false detection bounding box and is eliminated.

As Proposed-II sophisticatedly involves Proposed-I and Faster R-CNN, it integrates the merits of both Proposed-I and Faster R-CNN while overcoming their disadvantages. Thus, a higher DR could be achieved at the cost of a smaller FDR, as demonstrated in the next section.

## 5. Experiments and Analysis

Next, we evaluated the Proposed-I and Proposed-II, which were described in Section 3 and Section 4, respectively. We first outline the experimental settings, then define evaluation metrics, and finally provide results for the ablation and comprehensive experimental simulations.

### 5.1. Experimental Settings

All experiments were run on a GPU server with 12 Intel^®^ Core™ i7-8700 CPU with 3.20 GHz, 31.3 GiB memory, two GeForce GTX 1080 Ti GPUs and an Ubuntu 16.04 LTS operating system. Python, TensorFlow and PyTorch were employed to implement Proposed-I and Proposed-II as well as the other CNN-based object detectors in the literature.

In the implementation, we randomly initialized all layers of the light-weight CNNs in Table 2 by drawing weights from a zero-mean Gaussian distribution with a standard deviation of 1, and used the adaptive moment estimation with $\beta_{1}=0.9$, $\beta_{2}=0.999$, and $\epsilon =1\times 10^{-8}$ to train the light weight CNNs. We deployed the Xavier initialization method to initialize all layers of VGG-16, and applied the min-batch stochastic gradient descent with momentum 0.9 to train VGG-16. In CNN model training, the cross entropy loss function was adopted for the light weight CNN, and that of entropy with $L_{2}$ regularization (scale = 0.0005) was set for VGG-16.

In addition, when the receptive field of CNN extended beyond the image border, the missing portions were filled with white pixels [255,255,255], attempting to retain the object size.

### 5.2. Evaluation Metrics

As introduced in [3,4], the DR (detection rate) and FDR (false detection rate) are generally adopted to evaluate the performance of insect pest counting. In essence, *DR* is equivalent to the metric of recall and FDR equals (1−precision). Recall, *R*, and precision, *P*, are defined as:

$$R=\frac{TP}{TP+FN},\;P=\frac{TP}{TP+FP},$$

where *TP*, *FN*, and *FP* denote the true positive, false negative and false positive values, respectively.

In our case, the TPs are determined as follows: as we pay more attention to counting accuracy than to locating performance, we did not develop a bounding-box regressor in our insect pest counter. Instead, we considered a detection bounding box *b* to be “correct” if $b.\mathrm{cls}=b_{\mathrm{gt}}.\mathrm{cls}$ and $\mathrm{iogt}(b,b_{\mathrm{gt}})\geq 0.5$, where $b_{\mathrm{gt}}$ denotes the ground-truth bounding box. If several “correct” detection bounding boxes exist, only the one with the highest $\mathrm{iogt}(b,b_{\mathrm{gt}})$ is chosen as the true positive while the others are treated as false positives.

As different approaches generally lead to various DRs and FDRs, it may be troublesome to assess the performance of given approaches. For instance, one method obtains *DR* = 0.85 at *FDR* = 0.2, and another achieves *DR* = 0.92 and *FDR* = 0.4, which are difficult to fairly compare. To tackle this issue, we then used the $F_{1}$ score as the evaluation metric, which is defined as:

$$F_{1}=\frac{2\times \mathrm{DR}\times (1-\mathrm{FDR})}{\mathrm{DR}+(1-\mathrm{FDR})}=\frac{2\times R\times P}{R+P}.$$

In evaluating the insect pest counting performance, the mean absolute error (*MAE*) and mean squared error (*MSE*) are generally used. They are defined as:

(2) $$\begin{array}{cc} MAE & =\frac{1}{N}\sum_{i=1}^{N}\left|z_{i}-\hat{z_{i}}\right|\\ MSE & =\sqrt{\frac{1}{N}\sum_{i=1}^{N}{\left(z_{i}-\hat{z_{i}}\right)}^{2}}, \end{array}$$

where *N*, $\hat{z_{i}}$ and $z_{i}$ denote the total number of test images, the insect pest number in each test image calculated via a certain method, and the true insect pest number in each test image, respectively.

### 5.3. Experimental Results

We first examined the effectiveness of different modules of Proposed-I via ablation experiments and explore the performance of different backbone networks. We then selected a CNN model with the best performance as the backbone network and conducted comprehensive simulations to compare our methods with the state-of-the-art CNN-based object detectors as well as insect pest detection methods, demonstrating the feasibility of Proposed-I and Proposed-II.

#### 5.3.1. Ablation Experiments on Different Modules

**Ablation Experiments on the Background–Foreground Classifier and Tune-up Boxes.** As mentioned in Section 3.2, we exploited a saliency map to mimic the mechanism through by humans select a candidate region, applied the background–foreground classifier to select candidate regions probably containing foreground objects, and imposed *k* tune-up boxes to refine the candidate regions and thus yield the final region proposals. As the center of an activation region constructed from the saliency map is used as the anchor, most irrelevant background regions (i.e., easy negatives) are removed, as shown in Figure 2 and Figure 3. Below, we mainly focus on assessing the effectiveness of the background–foreground classifier and the tune-up boxes via ablation experiments.

In the simulation, we considered three cases: (1) removing the tune-up boxes; (2) removing the background–foreground classifier; and (3) removing both the tune-up boxes and the background–foreground classifier, which is essentially equivalent to the method in [24]. For each case, only the concerned part in Proposed-I is eliminated while the others remained unchanged, and the CNN model in Table 2 was employed as the backbone network of Proposed-I. Each modified network was then trained and tested accordingly. Table 3 presents the results in terms of DR, FDR and $F_{1}$-score.

We found that, compared with Case 3, Case 1, which adds the background–foreground classifiers, produced a significantly improved $F_{1}$ score, which indicates that the multi-stage architecture contributes to the effective decrease in FDR. Similar results were also observed for the comparison between Cases 2 and 3, where the tune-up boxes module was inserted for Case 2. These two cases thus demonstrate well the effectiveness of the background–foreground classifier and tune-up boxes developed in our study.

The comparison between Proposed-I and Cases 1–3 showed that integrating both the background–foreground classifier and tune-up boxes considerably improved the $F_{1}$ score further. As Case 3 is essentially the method in [24], this result also demonstrated that Proposed-I actually produced a significant improvement of up to 27.7%(=(75.6 − 59.2)/59.2) over the state-of-the-art insect pest detection method in [24].

**Ablation Experiments on the INMS Algorithm.** By considering the relationship between detection bounding boxes, we improved the conventional NMS (non-maximum suppression) via a merging strategy, as described in Section 3.2.3. To demonstrate the effectiveness of the INMS (improved NMS) on the suppression of false and non-optimal detection bounding boxes, we modified the Proposed-I by replacing the INMS with the conventional NMS and then compared it with the original one, where the CNN model in Table 2 served as the backbone network for creating an acceptable trade-off between performance and computational complexity. Table 4 provides the simulation results. INMS is more robust to the false positives and thus helps the Proposed-I achieve remarkable improvements in accuracy.

**Ablation Experiments on the CNN Architecture.** In our work, we adopted the widely used CNN to build the background–foreground and object classifiers. Given the trade-off between performance and computational complexity, we explored the lightweight CNN in Table 2 and VGG-16 [11] via ablation experiments on the CNN architecture. The experimental results are provided in Table 5. We observed that using a deeper network enabled Proposed-I to effectively increase the DR while decreasing the FDR. As the computational complexity of VGG-16 is acceptable, we thus chose VGG-16 as the CNN model for the backbone network of our proposed scheme.

#### 5.3.2. Comprehensive Assessment of the Proposed Scheme

To further examine Proposed-I and Proposed-II, we compared them with the state-of-the-art CNN-based object detectors including SSD [18], R-FCN [16], Faster R-CNN [15] and YOLOv4 [38]. We also conduct a comparison with the state-of-the-art insect pest detection method in [24], which is denoted as LIU for notational convenience. For fair comparison, VGG-16 served as their backbone networks. The experimental results are shown in Table 6.

Proposed-I achieved the smallest FDR with feasible DR whereas the Faster R-CNN had the highest DR at the cost of a relatively large FDR. Proposed-I attained a larger $F_{1}$ score than SSD, R-FCN, Faster R-CNN, YOLOv4 and LIU, which well demonstrates the feasibility and effectiveness of Proposed-I. YOLOv4 being inferior to Faster R-CNN may be attributed to the overfitting in YOLOv4.

We investigated the insect pest counting performance. SSD, R-FCN, Faster R-CNN, YOLOv4, LIU, Proposed-I and Proposed-II were first employed to detect diamondback moths in each test image; the detected diamondback moths were then counted. The MAE and MSE in Equation (2) with respect to the true number of diamondback moths were finally calculated via Equation (2) and the results are shown in Table 7. We observed that Proposed-I and Proposed-II achieved a significant improvement in terms of MAE and MSE compared with the other methods. Proposed-II, incorporating the dual networks, produced somewhat better results than Proposed-I, which was expected since Proposed-II simultaneously exploits the benefits of Proposed-I and Faster R-CNN.

### 5.4. Discussion

**Figure 6:**
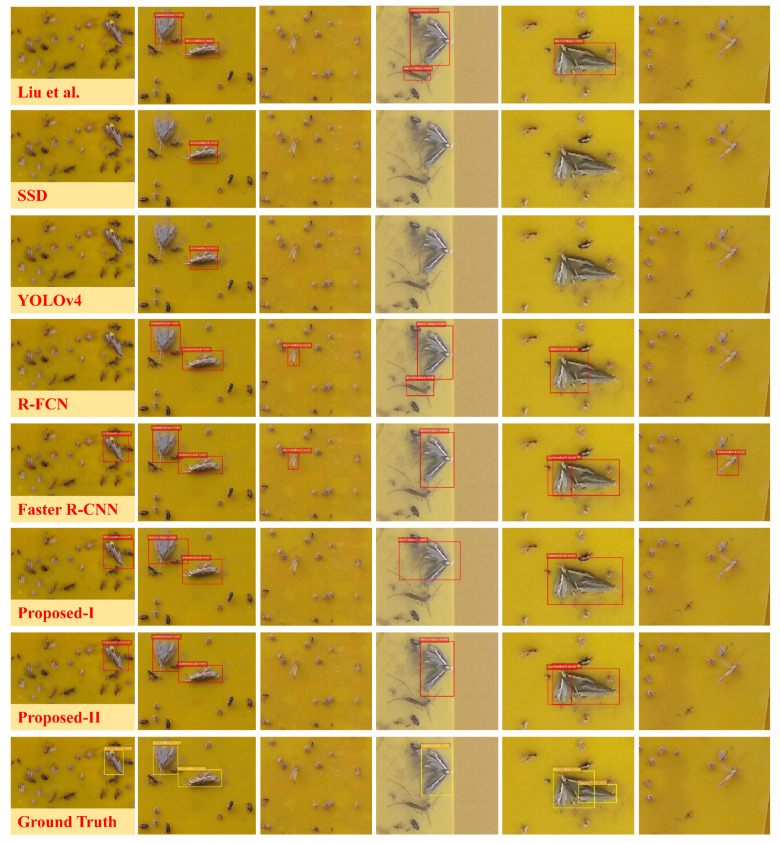
Some examples of the detection visualization results on the testing set. Rows 1–6 are results from LIU, SSD, YOLOv4, R-FCN, Faster R-CNN, Proposed-I, Proposed-II and ground-truth, respectively, and Columns 1–6 are different insect pest images.

Figure 6 visualizes detection and classification results for LIU, SSD, YOLOv4, R-FCN, Faster R-CNN, Proposed-I and Proposed-II. We found that Proposed-I and Proposed-II deal with background samples more effectively using the saliency map and merging strategy. With the saliency map, a large number of easy negatives are eliminated so that the object classifier is able to powerfully handle the hard negatives. With the merging strategy, the confidence in any true positive is increased; consequently, the redundancy eliminator does not perform arbitrary suppression. As a result, significant improvement over the state-of-the-art methods was achieved.

Proposed-I, however, performed unsatisfactorily when insect pests were close to each other because the proposed scheme relies heavily on the saliency map, which does not handle occlusion and overlap situations well. To alleviate this problem, we combined Proposed-I with Faster R-CNN to yield Proposed-II.

Although Proposed-II is promising, it is somewhat time-consuming and the performance can be further improved. For example, instance segmentation [43] may be applied to attain a better solution. In addition, the greedy merging strategy is sub-optimal, which may lead to false detections. To remedy this, contextual information [44] and a graph neural network can be employed to better re-score the confidence of the detection bounding boxes.

## 6. Conclusions

In this paper, we present an insect pest counter using a saliency map and INMS that is composed of four modules, that is, the region proposal generator, object classifier, INMS and insect pest number computation. The region proposal generator exploits a saliency map to effectively discard anchors corresponding to easy negatives, employs a CNN-based background–foreground classifier to select candidate regions that probably contain insect pest objects, and imposes *k* tune-up boxes on the center of candidate regions to generate region proposals. The object classifier uses a CNN model to determine the category of each region proposal. The INMS is improved from the conventional NMS by considering the relationship between detection bounding boxes, which accurately eliminates the false or sub-optimal detection bounding boxes. The insect pest number computation simply accumulates the number of the final detection bounding boxes. To tackle the issue of the missing detection of objects close to each other, we further integrated the proposed insect pest counter with conventional Faster R-CNN to construct a new dual-path-based insect pest counter. Extensive experimental simulations showed that the proposed two insect pest counters perform significantly better than state-of-the-art methods, demonstrating the feasibility and effectiveness of the proposed two insect pest counters.

In this study, we mainly focused on the diamondback moth, one of the key insect pests in vegetable fields. In our future research, we will extend this work to other key insect pests in vegetable fields, for example, thrips, Bemisia tabaci, and so forth. In addition, as our region proposal generator yields the activation region by forming connected graphs from the saliency map, it may group objects close to each other in a region, resulting in missing detections. Thus, accurately detecting objects close to each other is a challenging problem deserving future research. The deep-learning-based instance segmentation may be a feasible method for addressing this problem.

## Figures and Tables

**Table 1:** Main abbreviations in the paper.

Abbreviations	Full Name
CNN	Convolutional neural network
NMS	Non-maximum suppression
INMS	Improved NMS
OverFeat	The object detection method in [13]
Faster R-CNN	The object detection method in [15]
VGG	The object detection method in [11]
R-FCN	The object detection method in [16]
YOLO	The object detection method in [17]
YOLOv4	The object detection method in [38], which is version 4 of YOLO
SSD	The object detection method in [18]
LIU	The insect pest detection method in [24]
Proposed-I	The proposed insect pest counter based on a saliency map and INMS, as described in Section 3
Proposed-II	Another proposed insect pest counter using the dual networks, as presented in Section 4
DR	Detection rate
FDR	False detection rate
MAE	Mean absolute error
MSE	Mean squared error

**Table 2:** Configuration of the lightweight CNN.

Layer	${\mathrm{CNN}}_{lw}$
$L_{1,\mathrm{conv}}$	(7, 2, 40)
$L_{1,\max-\mathrm{pool}}$	(2, 2)
$L_{2,\mathrm{conv}}$	(5, 2, 60)
$L_{2,\max-\mathrm{pool}}$	(2, 2)
$L_{3,\mathrm{conv}}$	(3, 2, 120)
$L_{3,\max-\mathrm{pool}}$	(2, 2)
$L_{4,\mathrm{full}}$	100
$L_{5,\mathrm{full}}$	50

**Table 3:** The ablation experiment (%) on the testing set for the background-foreground classifier and tune-up boxes. ${\mathrm{CNN}}_{lw}$ was used as the backbone network.

Case	FDR	DR	$F_{1}$-Score
Case 1	27.3	69.6	71.1
Case 2	38.2	**79.3**	69.5
Case 3	45.6	65.0	59.2
Proposed-I	**22.0**	73.4	**75.6**

**Table 4:** The ablation experiments (%) on the testing set for INMS. ${\mathrm{CNN}}_{lw}$ was taken as the backbone network; “−” and “+” denote the elimination and employment of a certain part, respectively.

Method	FDR	DR	$F_{1}$-Score
Proposed-I − INMS + NMS	34.3	66.2	65.9
Proposed-I	**22.0**	**73.4**	**75.6**

**Table 5:** The ablation experiment (%) on the testing set for the CNN architecture. X⋄Y indicates that the backbone network of *X* is *Y*.

Method	FDR	DR	$F_{1}$-Score
Proposed-I ⋄ ${\mathrm{CNN}}_{lw}$	22.0	73.4	75.6
Proposed-I ⋄ VGG-16	**6.5**	**85.7**	**89.4**

**Table 6:** Performance comparison (%) on the testing set for SSD, R-FCN, Faster R-CNN, YOLOv4, LIU, Proposed-I and Proposed-II, where values in bold and underlined denote the best and second best results, respectively. The backbone networks of all the detectors were VGG-16.

Method	FDR	DR	$F_{1}$-Score
SSD	8.5	40.9	56.5
R-FCN	41.2	70.5	64.1
Faster R-CNN	22.8	95.8	85.5
YOLOv4	1.9	43.9	60.7
LIU	28.1	71.3	71.6
Proposed-I	6.5	85.7	**89.4**
Proposed-II	12.8	89.5	88.3

**Table 7:** Comparison of insect pest counting performance on the testing set for SSD, R-FCN, Faster R-CNN, YOLOv4, LIU, Proposed-I and Proposed-II, where values in bold and underlined denote the best and second best values, respectively.

Method	MAE	MSE
SSD	0.117	0.340
R-FCN	0.147	0.418
Faster R-CNN	0.059	0.263
YOLOv4	0.117	0.390
LIU	0.079	0.281
Proposed-I	0.047	0.224
Proposed-II	**0.040**	**0.216**
